# Conservative treatments of bone marrow lesions

**DOI:** 10.1002/jeo2.70151

**Published:** 2025-04-04

**Authors:** Luca Andriolo, Alessandro Sangiorgio, Massimo Berruto, Henning Madry, Giuseppe M. Peretti, Massimo Varenna, Beer Yiftah, Stefano Zaffagnini, Giuseppe Filardo

**Affiliations:** ^1^ Clinica Ortopedica e Traumatologica 2, IRCCS Istituto Ortopedico Rizzoli Bologna Italy; ^2^ Service of Orthopaedics and Traumatology, Department of Surgery EOC Lugano Switzerland; ^3^ U.O.C. 1st Orthopedic Clinic, ASST Gaetano Pini‐CTO Milan Italy; ^4^ Center of Experimental Orthopaedics Saarland University Homburg Germany; ^5^ E.U.O.R.R. Unit, Department of Biomedical Sciences for Health, IRCCS Orthopedic Institute Galeazzi University “La Statale” Milan Italy; ^6^ Bone Diseases Unit, Department of Rheumatology and Medical Sciences ASST Gaetano Pini‐CTO Milan Italy; ^7^ Shamir Medical Center Be'er Ya'akov Israel; ^8^ Faculty of Biomedical Sciences Università della Svizzera Italiana Lugano Switzerland

**Keywords:** bone bruise, bone marrow lesion, cartilage regeneration, conservative treatment

## Abstract

**Purpose:**

Bone marrow lesions (BMLs) of the knee are a common magnetic resonance imaging finding and are present in a wide range of pathologies, including traumatic contusions and fractures, following cartilage surgery alterations, osteoarthritis, transient BMLs syndromes, subchondral insufficiency fractures of the knee and spontaneous osteonecrosis of the knee. Regardless of their aetiology, clinical management may prove challenging. This review focuses on the conservative treatment approaches to manage patients affected by knee BML, thanks to the contribution of field experts.

**Methods:**

Experts from around the globe were involved in performing a review on the most used conservative treatment strategies to address BMLs, trying to summarize the available evidence from the most popular first‐line treatments while documenting their applications and results for the different BML aetiologies.

**Results:**

Positive results were documented for unloading knee braces, external shockwave therapy, hyperbaric oxygen therapy, pulsed electromagnetic fields therapy and bisphosphonates. Nonetheless, the analysis of the scientific literature documented a scarce number of publications specifically addressing the knee joint, with even less evidence when it comes to the results for the different aetiologies of BMLs.

**Conclusion:**

The management of BMLs is challenging, and many factors influence clinical and radiological outcomes. This paper summarized the evidence on conservative treatments for knee BMLs. Although showing promising results, conservative options still need to be fully investigated. Open questions to be addressed concern treatment duration, BML stage and overlapping with concomitant therapies. Further studies are needed to identify the best first‐line conservative approach or treatment combination based on each BML aetiology.

**Level of Evidence:**

Level V: expert opinion.

**Abbreviations**
AVN: avascular necrosis
BML: bone marrow lesion
BP: bisphosphonate
CRPS: complex regional pain syndrome
ESWT: extracorporeal shock wave therapy
HBOT: hyperbaric oxygen therapy
IGF‐I and IGF‐II: insulin‐like growth factors I and II
MRI: magnetic resonance imaging
MSC: mesenchymal stem cell
NSAID: non‐steroidal anti‐inflammatory drug
OA: osteoarthritis
PEMF: pulsed electromagnetic field
RMO: regional migratory osteoporosis
SIFK: subchondral insufficiency fractures of the knee
TGF‐ß: transforming growth factor beta
TOH: transient osteoporosis of the hip

## INTRODUCTION

Bone marrow lesions (BMLs) of the knee are a common magnetic resonance imaging (MRI) finding. A BML is defined as an alteration of the bone marrow signal intensity, with high signal on fluid‐sensitive sequences (T2/proton density with fat suppression and Short tau inversion recovery) with or without low T1‐weighted image signal. BML is present in a wide range of pathologies [47]: traumatic BMLs are associated with acute direct or indirect trauma (i.e., bone contusions) or with subacute progressive lesions as a result of overload (i.e., stress fractures and repetitive microtrauma occurring during physical activity); BML syndromes (such as complex regional pain syndrome [CRPS], transient osteoporosis of the hip [TOH] or regional migratory osteoporosis [RMO]) are not associated with trauma and are considered as reversible BML conditions with complex and sometimes unknown aetiology; post‐cartilage surgery imaging alterations, characterized often by an altered joint load and by bone tissue alterations; BMLs related to the osteoarthritis (OA) process represent an example of reversible lesions [29, 103]; subchondral insufficiency fractures of the knee (SIFK) [107] can be reversible, but can also progress to secondary irreversible osteonecrosis; true osteonecrosis, further divided in avascular necrosis (AVN) and primary osteonecrosis [56]. Based on histological evidence [99, 100], SIFK has been proposed as the true aetiology of osteonecrosis (previously known also as spontaneous osteonecrosis of the knee), considering AVN with its ischaemic aetiology as a different pathology. BMLs showed by MRI results from various aetiologies affecting the subchondral bone and reflect a multitude of histopathological findings: in bone contusion, (micro) fractures of the subarticular spongiosa with osteocyte necrosis and empty lacunae, haemorrhage and oedema; in OA, increased thickness of the subarticular trabeculae, microcracks, micro‐oedema, microbleeding within the subchondral region and subchondral bone cysts; in SIFK and SIFK‐related osteonecrosis necrotic tissue, subsequently removed and replaced by newly formed trabecular bone in the subarticular spongiosa by creeping substitution, a sclerotic rim at the interface between the necrotic zone and the surrounding viable subchondral bone [47]. BMLs may also be present in tumours and leukaemia, as well as in physiological conditions like disuse, which will not be considered in the present manuscript [47]. Each pathological pattern is determined by different aetiologic factors (including major or minor trauma, bone tissue alterations, altered joint load distribution, coagulopathies and hormonal alterations) which may act alone or synergically in determining the BML [5, 47].

These pathologic patterns present different prognoses; thus, a careful diagnosis is mandatory to address them with the proper treatment. In fact, BML may sometimes be the treatment target, and different treatments may be required for different aetiologies. Even though no treatment algorithm is available to choose between surgical or conservative treatment for the different patterns, nor conclusive evidence about the results of the different conservative treatments, some general considerations can be made. For example, traumatic BMLs are generally managed with unloading, while BMLs occurring in the course of OA can be treated with both conservative and surgical management. SIFK‐related osteonecrosis may benefit from various forms of treatment, like physical therapies, drugs or even surgery, based on symptoms and disease stage. The decision is largely based on the aetiology, size, and grade of the affected area [73, 78], considering that lesions bigger than 5 cm^2^ usually lead to collapse and require prosthetic arthroplasty [7, 72], medium‐size lesions (3.5–5.0 cm^2^) may or may not regress, and small lesions (typically <3.5 cm^2^) usually regress with non‐surgical management [44]. The conservative approach includes treatment with non‐steroidal anti‐inflammatory drugs (NSAIDs), analgesics and protected weight bearing for three to eight months, as well as more targeted pharmaceutical approaches, based on patients' symptoms and radiological control [44, 101]. Other areas of non‐operative management currently being studied are pulsed electromagnetic field (PEMF) therapy and hyperbaric oxygen therapy (HBOT). The conservative management of BML is challenging, with many possible treatment approaches and many factors influencing the clinical outcome. To date, clear indications about the best treatment for the different BML patterns are not yet available. The state‐of‐the‐art conservative treatments for BML of the knee are discussed in the next paragraphs.

## UNLOADING: A NON‐INVASIVE, BIOMECHANICAL THERAPY

Although BMLs may be self‐limiting, conservative therapies aim at shortening the usual course of the disease, while avoiding invasive surgeries. However, the most successful conservative measure has not been identified. The treatment of BMLs is currently not standardized, making it difficult to retrieve data that can be compared in a reliable way [28]. With regard to the many non‐standardized measures, the current literature shows promising results on the improvement in the clinical course of BML, but further large multicentre randomized controlled trials, as well as standardized radiological and clinical scores, are warranted to acquire evidence‐based recommendations on the best therapeutic algorithm [71].

Unloading knee braces has been proposed as a possible first‐line treatment strategy. They consist of a frame of lightweight materials, such as carbon fibres or aluminium, offering a thigh support above the knee and a calf support below, connected by hinges. Straps on the side help secure the brace while offering an adjustable fit. The main function of a brace is to redistribute weight‐bearing loads within the knee joint by applying corrective forces. This helps realign the knee joint, offloading pressure from the damaged area and relieving symptoms. Knee bracing improves the biochemical properties of the articular cartilage by reducing the extent of oedema while increasing collagen and proteoglycan concentration. The use of an unloading knee brace helps keep antero‐posterior translation while reducing abduction‐adduction rotation [37], with a mechanical effect overall reducing tibio‐femoral stress. Although unloading strategies have shown overall clinical benefits, their effectiveness in improving BMLs is still unclear. Few studies have addressed this treatment option, with conflicting results [82]. A recent case series documented positive results in terms of clinical outcome and MRI parameters such as thickness or the extent of the damaged cartilage in patients treated with unloading bracing for knee unicompartmental OA [9]. A randomized controlled trial addressed knee bracing for the treatment of patello‐femoral OA [18], finding a significantly lower knee pain and a reduction in BML extent compared to the no brace group, suggesting a promising effect of the knee brace in relieving BML and OA‐associated symptoms. Nonetheless, another trial found no differences in an unloading knee brace compared to the usual physical therapy in patients with medial compartment OA [39]. The effects of knee bracing have also been investigated in post‐cartilage surgery patients, with a randomized study documenting a significant mid‐term increase in cartilage repair tissue thickness in patients treated with microfractures for isolated chondral defects [48].

In conclusion, despite the promising results, the evidence supporting the use of unloading knee bracing is still very limited. The major issue is the small number of trials addressing this conservative option and the differences in load distribution among the various braces employed [14, 62, 75]. In addition, limitations of the available literature include the lack of analysis for possible confounders such as concomitant physical therapy protocols [104], placebo effect, patients' compliance [88], and painkiller drug intake [17]. Future studies focusing on BMLs should clarify the effects of unloading knee bracing as a valid conservative strategy for these pathologies, taking into account the abovementioned confounders, alone or in combination with the other conservative treatment approaches.

## EXTRACORPOREAL SHOCKWAVE THERAPY

Another non‐surgical option largely used in clinical practice is extracorporeal shock wave therapy (ESWT), which proved effective in the conservative treatment of various orthopaedic conditions. Particularly, ESWT has been associated with BML reduction in the early stages of AVN [77]. ESWT provides a mechanical stimulation that has an effect on the healing processes while helping to normalize the impaired bone homoeostasis occurring during BML. There are studies in the literature showing how the ESTW influence the cellular expression of insulin‐like growth factor I (IGF‐I), transforming growth factor beta (TGF‐β)1 and other cytokines [98]. Furthermore, the release of growth factors may contribute to the neo‐vessel formation alongside increased cell growth and collagen production [95]. Finally, ESWT promotes tissue regeneration by stimulating osteoblasts while suppressing osteoclast activity [23], and it may also contribute to normalizing damaged blood vessels and joint metabolism [53].

ESWT has been investigated as a treatment option in the conservative management of BML patients, showing promising results in pre‐clinical studies and in BML located in joints other than the knee [21, 22]. An in vivo study assessed the efficacy of ESWT in an OA model of mice knees, documenting significant improvements in subchondral bone repair, especially in the medial tibia and medial femoral condyle. The immunohistochemical analysis documented an increased cartilage proliferation and reduced vascular invasion through the observation of cluster of differentiation 31, a marker for angiogenesis [96]. A retrospective MRI study on 67 patients suffering from BML caused by femoral head osteonecrosis showed significant pain relief and functional improvement after ESWT treatment, alongside a significant reduction in the BML area extent [106]. Another case series by Sansone et al. concluded that ESWT is a safe and effective treatment for knee BMLs, and it can accelerate the functional recovery of patients [77]. In this publication, BML size significantly correlated with knee pain intensity. Additionally, the MRI evaluation documented a significant reduction of BMLs at follow‐up, demonstrating a positive correlation with the improvement in the clinical questionnaires. Finally, a comparative study documented an 86% reduction in BML area at long‐term follow‐up after ESWT in patients suffering from knee medial compartment BMLs, while the reduction in the control group was 41%. In addition, clinical improvement was noticed in the third month after starting the treatment [77].

The evidence from these investigations has been summarized in recent literature reviews addressing the effectiveness of ESWT in the treatment of BMLs, comparing pain and functional improvements to other conservative measures and surgery [28, 38, 53]. Although limited by the heterogeneity and the overall low quality of the included studies, ESWT showed promising results in reducing BML extent on MRI and BML‐related pain and functional limitation. However, as previously underlined for unloading knee braces, the influence of the course of natural history and the placebo effect cannot be excluded as concomitant factors in the reduction of BML area and in symptom improvement over time, especially at longer follow‐ups. Moreover, it remains unclear if the positive effects observed after ESWT treatment could be equally obtained for all the possible aetiologies of BMLs. More studies should clarify the real potential of ESWT by focusing on each specific pathological condition causing BMLs, also clarifying the most suitable energy level to be applied, frequency of treatment, concomitant therapies and the total number of treatments used to optimize ESWT treatment regimen.

## HYPERBARIC OXYGEN THERAPY

The non‐surgical management of SIFK‐related osteonecrosis and other BML pathologies benefits from a combination of pharmacological and non‐pharmacological modalities, like protected weight bearing, analgesics and NSAIDs, if tolerated. Resolution of symptoms can be expected in 89% of patients with pre‐collapse disease and no articular changes on plain radiographs [86, 101]. To further enhance the success rate of conservative management, different approaches are being studied, including the field of physical therapies. Even though evidence on the optimal physical therapy intervention is controversial [30, 46], overall adverse events are uncommon, and not severe enough to prevent participants from continuing treatments, which explains their wide use in the clinical practice, regardless of the unclear evidence on which approach may provide the best results [97]. In this context, HBOT has been applied mostly to address OA and osteonecrosis.

During a standard HBOT session, the patient is placed inside a treatment chamber breathing 100% oxygen intermittently. The pressure protocols are usually set between 1.5 and 3.0 atmosphere absolute (ATA), with an exposure time of 1–2 h, depending on the indications. According to Boyle's law, as the pressure in a chamber is elevated, the volume of the same mass of gas is reduced, and, therefore, there are more molecules per space. According to William Henri's law, ‘The solubility of a gas in a liquid is directly proportional to the partial pressure of the gas above the liquid’. Breathing 100% oxygen under pressure causes the oxygen to diffuse into the blood plasma, increasing haemoglobin saturation. Thus, oxygen‐rich plasma is able to travel and diffuse many times further into the tissue. The pressurized environment helps to reduce swelling and discomfort, while providing the body with at least 10 times its normal supply of oxygen to help repair tissue damaged by the original occlusion. Furthermore, an in vivo study confirmed that HBOT increases hypoxia‐inducible factor 1 production, thus promoting and sustaining tissue healing [83]. HBOT also forces more oxygen into the tissue, encouraging the formation of new blood vessels [102]. As these new blood vessels develop, even more oxygen is delivered to the affected area [36].

HBOT has been suggested to provide beneficial effects to stage I AVN of the femoral head [74]. In a retrospective study, up to 81%–88% of patients who received HBOT showed complete healing on MRI as compared with 17% in the untreated group. At 11.1 ± 5.1 years of follow‐up, joint survivorship accounted for 93% [49]. Other investigations concluded that HBOT played an important role in treating Stages I and II osteonecrosis of the femoral head, with BML improvement and a concomitant decrease of inflammatory mediators such as TNF‐α and interleukin‐6 [13]. Nonetheless, the body of evidence regarding HBOT treatment is still limited by the few number of articles on this topic, as documented by a literature review [87], which highlighted the heterogeneity and the overall weak methodology of the published studies.

More recently, HBOT has also been used to treat BMLs located in the knee joint. A retrospective study investigated the application of HBOT in patients suffering from SIFK‐related osteonecrosis [12], documenting a significant improvement of knee BML treated with HBOT, with an MRI follow‐up of 7 years. Another recent comparative study documented a statistically significant 35% knee BML extent reduction in patients receiving treatment with acetylsalicylic acid, bisphosphonates (BPs) and HBOT compared to a non‐significant 5% area reduction in patients who received the pharmacological treatment only [70]. In these two publications, a similar HBOT protocol was employed, with sessions performed once a day, 5 days a week at 2.5 ATA, but with a difference in treatment duration (1 month vs. 1.5 months followed by a 2‐month break and a second cycle on 1 month) between the two studies.

Further studies are needed to understand the real potential and the best indications for HBOT for the treatment of BML pathologies. The promising results reported are based only on few publications with a limited sample size, thus preventing the possibility of drawing solid conclusions. In addition, HBOT literature suffers from the same limitations encountered for unloading braces and ESWT, and more studies are needed in order to clarify the impact that concomitant therapies, treatment duration, BML extent and aetiology have on the clinical and radiological evolution of BMLs treated with HBOT.

## ELECTROMAGNETIC FIELDS THERAPY

The use of PEMFs was studied as a treatment of the osteonecrosis of the femoral head [52] and in the knee. Moreover, it has been proposed as a possible new approach to reduce knee OA progression [31]. The use of exogenous electrical currents, administered at specific amplitude and frequency, showed positive effects on bone formation, bone graft incorporation and bone repair in both in vitro and in vivo models [2, 54]; it was also shown to reduce knee osteoarthritic lesion progression in guinea pigs [31]. Preclinical experiments highlighted that PEMF stimulation with specific exposure parameters may exert a chondroprotective effect on articular cartilage by increasing proteoglycan synthesis and counteracting the catabolic activity of pro‐inflammatory cytokines, together with positive effects also by inhibiting subchondral bone sclerosis, particularly in early OA stages [46]. Moreover, this treatment is reported to produce anti‐inflammatory [40] and bone‐healing effects [16, 33], thus reducing the production of free radicals and stimulating the osteogenic activity of osteoblasts [45]. In addition, PEMFs have been shown to decrease parathyroid hormone receptor activity on osteoblasts and reduce the lysosomal content of osteoclasts thereby suppressing bone resorption and overall increasing bone mass [54].

PEMFs have been proposed to exert their effects based on the following three concepts: Wolff's law, the piezoelectric effect and streaming potentials [85]. Wolff's law states that bones respond to the mechanical loads under which they are placed: compression results in osteogenesis on the side compressed and simultaneous resorption on the contralateral side [32]. This occurs via a process called mechanotransduction whereby mechanical signals are transformed into biochemical ones [40]. The piezoelectric effect describes the phenomenon where certain materials demonstrate an ability to generate negative and positive potentials when subjected to mechanical strain. In bone, the piezoelectric nature of hydroxyapatite (HA) and collagen results in a negative potential generated during compression and a positive potential when the stress is relieved. Notably, the piezoelectric effect is reversible, hence the mechanical stress can be induced with the application of an electric field [33]. In cartilage, streaming potentials refer to the movement of positively charged ions across negatively charged proteoglycans during mechanical stress, generating an electric current that may stimulate chondrocytes [45]. Therefore, a possible PEMF application is the induction of a mechanical strain via the converse piezoelectric effect, thus inducing osteogenesis via Wolff's law, as well as chondrocyte stimulation [85]. The potential beneficial effects on the subchondral bone [31] and the ability to reduce inflammatory processes at the knee [16] explain the rationale for the treatment of BMLs.

The use of PEMFs in early‐stage SIFK‐related osteonecrosis was shown to reduce knee pain significantly (in 75% of cases) and necrosis area (in 85% of cases) already at the 6‐month follow‐up [57]. This treatment increased knee function with good‐to‐excellent functional results in 75% of cases, preserving 86% of knees from prosthetic surgery at the 24‐month follow‐up. These results are comparable to those obtained by PEMF stimulation in the treatment of osteonecrosis of the femoral head [59]: the treatment was able to preserve 80% of patients from total hip replacement at the 28‐month mean follow‐up. In addition, a systematic review of 10 articles describing the results of PEMFs for femoral head AVN reported that early stages showed the best responses, with improvements in both clinical and radiographic parameters [4]. PEMFs were demonstrated to be effective also in talus BMLs [58] with improvement of the clinical scores and resolution of the BML at 3 months MRI in five of the six patients. The necrotic area reduction in 85% of patients reported for PEMFs in the treatment of SIFK‐related osteonecrosis [57] appears higher than the rate reported for classic conservative treatments [50], where a resolution of BML at short‐term follow‐up has been described in 60% of patients with Stages I and II osteonecrosis treated with functional discharge and non‐steroid analgesics.

A notable limitation of these studies is the lack of comparison in terms of pain outcomes, hence making it difficult to ascertain the actual degree of clinical improvement. However, Aaron et al. found that more patients who received PEMF alone experienced less pain than patients who received core decompression alone [1]. This is the only study that directly compared the outcomes of PEMF therapy to a widely accepted treatment approach, and it showed a clear advantage of PEMF over core decompression. These findings show that PEMF therapy is a promising technique. Given its non‐invasive nature and potential to stop or reverse the disease process, PEMFs are an interesting area of research, especially for the management of early‐stage disease, although more studies are needed to confirm the potential and indications. Finally, the technique's adoption is partially hindered by the fact that its application is generally cumbersome and requires significant compliance from the patients. The devices often require long hours of use for many months and precise placement of the coils [2, 8, 19, 52, 59].

## PHARMACEUTICAL TREATMENT

In the last years, articles on increasing the level of evidence were published about the efficacy of bone‐targeting drugs, such as BPs and prostacyclin, in the treatment of musculoskeletal disorders characterized by BMLs. They act on different bone targets and, therefore, may interact with different steps of the etiopathological patterns of BMLs.

Prostacyclin (whose most studied analogue is Iloprost; Schering AG) has pharmacokinetic effects leading to better perfusion in tissues with a critical blood supply. In fact, it induces vasodilation [35], reduces capillary permeability, inhibits platelet aggregation and diminishes the concentration of free oxygen radicals and leukotrienes [3, 15, 26, 27]. It is unclear if the pain relief and reduction of BML during and after Iloprost application are primarily based on a normalization of intraosseous pressure or interactions with local leukotrienes and cytokines [43]. Nonetheless, the effects of Iloprost in treating BML were proved by several clinical studies at short‐term follow‐up, with lesion regression and symptom improvement [60, 61], although with poorer results in advanced stages [43].

**Figure 1:**
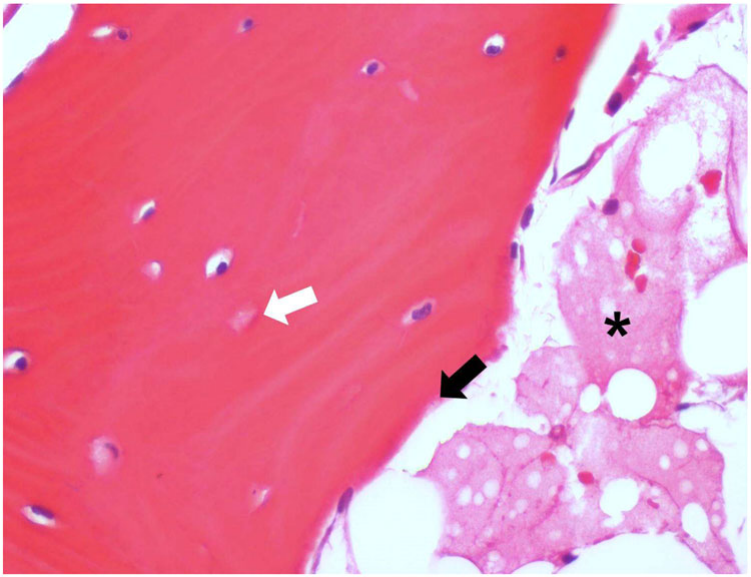
Histological specimen of a complex regional pain syndrome in the astragalus. A higher magnification histology image of a bone lamella in hematoxylin–eosin (H&E) staining. Interlamellar spaces are occupied by amorphous material, weakly eosinophilic (representing liquid/oedema), in place of the physiological adipose tissue (black asterisk). Lining is partially interrupted, in the absence of osteoblasts (black arrow). Dilatation of some osteocytic lacunae is observed; some others are empty, with indirect signs of osteocytic necrosis (white arrow). (H&E; 40x).

On the other hand, BPs inhibit osteoclast activity and consequently bone resorption. The rationale for the use of BPs in the treatment of BMLs and early, pre‐collapse stage osteonecrosis is based on the assumption that structural bone failure is the result of resorption of necrotic bone during revascularization before new bone has been formed. It can be hypothesized that if accelerated bone resorption could be reduced during the revascularization process until sufficient new bone has been formed, structural failure could be avoided. Interestingly, some studies proved the efficacy of BPs also in diseases which are not characterized by an increased osteoclast activity, but by BML linked to a microcirculation disorder, as evidenced by histopathological studies showing an amorphous, weakly eosinophilic infiltrate related to a transudate (Figure 1). It is likely that this peculiar pharmacokinetic is not related to an enhanced local bone turnover, as often erroneously deemed, while it can be explained by the effect on the lining cells and modified osteoblasts overlaying the trabecular bone surface. Lining cell disappearance following a microcirculation impairment uncovers a number of available binding sites at the HA crystal level. Due to this high local concentration, BPs can inhibit the local inflammatory infiltrate, detected at the disease site by radiolabelled leucocyte scintiscan studies, therefore inducing leucocyte necrosis by their pro‐apoptotic properties, mostly active on macrophagic population. Furthermore, as mentioned above, BPs could act as effective analgesics by reducing the number of cells with an anaerobic metabolism thus counteracting the lowered pH responsible for pain and possibly HA chemical dissolution. Consistently, a fast and significant improvement in bone densitometry is usually observed [93].

**Figure 2:**
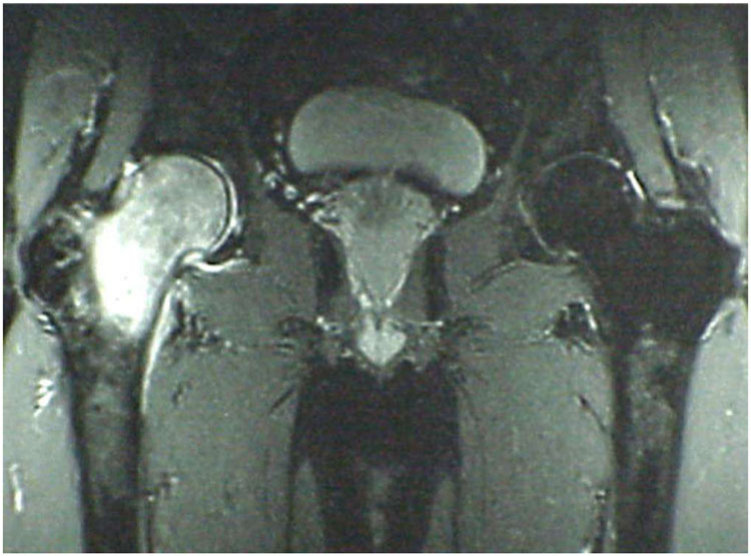
Coronal view with T2‐weighted fat‐suppressed MRI shows a high and homogenous hyperintense signal of head and femoral neck, saving trochanter, in the absence of focal abnormalities. The cortical profile is maintained, particularly the upper polar region remains spherical. No signal alterations on the acetabular side. A small intra‐articular effusion is present (1.5 T scanner; Magnetom Espree, Siemens). MRI, magnetic resonance imaging.

In disorders such as the early stages of CRPS, TOH (Figure 2) or RMO, molecules belonging to the BP family provide the most rewarding evidence of efficacy, as long as administrated in a short time from the onset and at high regimens by intravenous route [89, 90, 91, 92, 93]. Talking about the efficacy of BPs in the treatment of OA, it could be useful to raise some assumptions. During the last few years, the extensive use of MRI has highlighted the role played by subchondral bone in the clinical and pathophysiological features of this disease. As shown in several studies, subchondral areas with the same MRI features of BML correlate with pain onset and intensity [105], anatomical progression of the disease [25] and can even be predictive of surgical joint replacement [84]. Over the last decades, studies about the possible therapeutic role of BPs in the treatment of OA showed results sometimes not consistent, probably due to differences in BPs' duration of use, dose and route of administration [20, 24]. Furthermore, these discrepancies are probably related to heterogeneous and often inadequate inclusion criteria adopted by several studies. Moreover, some molecules used in these trials had a low efficacy profile, and some routes of administration (i.e., oral intake) failed to reach an adequate local concentration necessary to exert a therapeutic effect.

**Figure 3:**
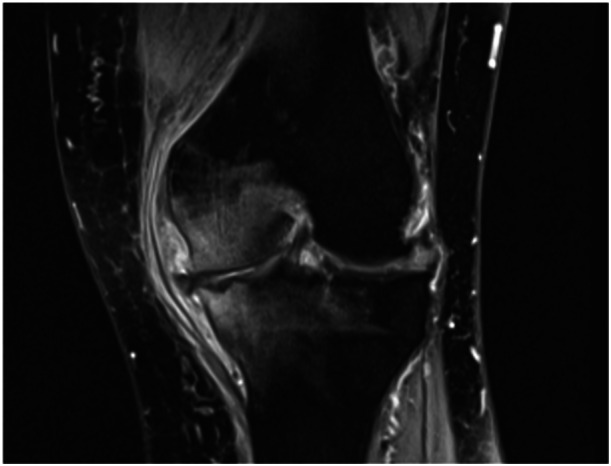
Coronal view in T2‐weighted fat‐suppressed magnetic resonance images demonstrating bone marrow oedema in the medial tibia and femur. Marked reduction of articular space, cartilage thickening, maceration and subluxation of the medial meniscus are present, indicating degenerative change of the knee. Lateral meniscopathy and chondropathy, less marked than the medial compartment, are shown. Soft tissue thickening with local synovial reaction that imprints and dislocates the capsuloligamentous compartment (1.5 T scanner; Magnetom Espree, Siemens).

To date, it is not possible to presume a pharmaceutical action of BP able to slow down or stop the anatomical progression of the disease. Notwithstanding, some animal models demonstrated a deranged skeletal turnover at the subchondral level in the early stages of experimentally induced OA, and bone changes appeared to influence cartilage metabolism by inducing a damage that worsens [11]. Consistently, the inhibition of bone turnover induced by BPs seemed to slow down the disease progression [34]. Convincing data related to analgesic properties were also gathered in real practice, consistent with a possible effect of BPs in reducing joint pain. This evidence is more easily observed not when a patient is suffering from chronic pain, but in the case of acute painful flare, which is more frequent and more intense in long‐standing disease, such as when MRI shows BMLs increasing in size (Figure 3). Taking into account the above, BPs can improve symptoms and functional measures of OA, provided these molecules are employed at proper dosages [94].

In conclusion, the role of a pharmaceutical approach by BPs in the treatment of BMLs nowadays represents a therapeutic strategy that can provide concrete benefits to patients as long as an accurate diagnosis of the underlying disease is made. Finally, the chance to obtain satisfactory and effective results goes necessarily through the choice of BPs by using proper molecules with adequate pharmacological potency and at dose regimens ensuring optimal achievement.

## A FUTURE CHALLENGE: THE CHICKEN OR THE EGG—THE BIOLOGY OF THE OSTEOCHONDRAL UNIT

The ‘chicken or the egg’ causality dilemma, applied to the concept of the osteochondral unit, raises the question of whether the articular cartilage or the subchondral bone plays the major underlying role in the development of pathologies such as OA or osteochondral defects [76]. This is key to understand how to properly manage most of the BMLs observed in clinical practice.

The articular calcified cartilage and the underlying subchondral bone form together a tight functional association [55]. The hyaline articular cartilage, which is an avascular and aneural tissue, consists of chondrocytes embedded in a dense extracellular matrix. This extracellular matrix contains mainly water (65%–80% of wet weight), Type II collagen and proteoglycans. It provides the joint with a low friction environment, its major features being lack of vascularisation, low cellularity and limited metabolic activity of mature chondrocytes. A tidemark can be seen on histological sections, separating the deep cartilage zone and the calcified cartilage layer. The calcified cartilage contains type‐X collagen besides the Type II collagen fibrils extending from the non‐calcified articular cartilage. It is closely connected with the subchondral bone plate by the cement line through three‐dimensional undulating interdigitations. The subchondral bone plate is a dense bony lamella, comparable to the cortical bone structures, separating calcified cartilage from the marrow cavity. Without a clear boundary, the subchondral bone plate merges into the subarticular spongiosa, a porous and metabolically active network of trabecular bone. These cavities are filled with bone marrow, containing mesenchymal stem cells (MSCs). The subchondral bone consists of osteoblasts, osteocytes and osteoclasts embedded in a mineralized matrix, which is capable of remodelling.

An osteochondral defect extends by definition into the subchondral bone. As a natural consequence, an osteochondral defect affects dissimilar tissues with divergent intrinsic healing capacities. For the cartilaginous repair tissue, an intrinsic inability to regenerate a native hyaline articular cartilage and its degeneration over time are the major problems. For the subchondral bone, several pathological features are associated with spontaneous osteochondral repair and with cartilage repair procedures, including the upward migration of the subchondral bone plate, formation of intralesional osteophytes, appearance of subchondral bone cysts and the generalized impairment of the osseous microarchitecture.

Focal osteochondral defects occur in the course of diseases such as osteochondritis dissecans and osteonecrosis, but also as a result of osteochondral fractures [63]. The opening of the subchondral bone cavities causes haemorrhage and activates inflammatory responses, filling the defect with a blood clot. Platelets within the clot release vasoactive mediators, growth factors and cytokines (among which TGF‐ß and platelet‐derived growth factor) that play a key role in supporting cell migration, proliferation, differentiation and matrix production [41]. In addition to these factors, the bone matrix also contains the bone morphogenetic proteins and IGF‐I and IGF‐II that have access to the lesion, where they play similar roles in initiating reparative responses [41]. Under such stimuli, MSCs from the voids in the subarticular spongiosa adjacent to the defect migrate into the lesion. They differentiate into chondrocytes and osteoblasts, initiating osseous and chondral repair responses depending on their spatial location, while the fibrin clot is being resorbed.

In the cartilaginous region of the osteochondral defect, MSC differentiation processes tend towards the chondrocyte phenotype in a defined sequence of cellular and molecular events [79]. Initially, MSCs initiate chondrogenesis by condensation through cell–cell interactions mediated by the expression of N‐cadherin [6]. After some days, the expression of this molecule is lost, and the cells acquire a spindle‐shaped, undifferentiated form with the presence of a fibrocartilaginous extracellular matrix consisting of Type I collagen and the expression of the PTH/PTHrP receptor that delays the progression of chondrogenic differentiation. Within 2 weeks, the PTH/PTHrP receptor is still detectable but the cells start to assume a typical rounded morphology, producing a matrix of Type II collagen and relatively high levels of proteoglycans [6, 42, 80]. Additionally, the expression of members of the family of DNA‐binding proteins SOX (sex‐determining region Y‐type high mobility group box) increases, especially SOX9 [6], a critical transcription factor for chondrocyte differentiation which activates the gene for Type II collagen and other cartilage‐specific genes [10]. After a few months, the repair tissue in this chondral region still contains chondrocyte‐like cells in a matrix of proteoglycans and Type II collagen, as well as Type I collagen, as an intermediate between hyaline and fibrocartilage. For example, the repair tissue does not exhibit the arcade‐like organization of the Type II collagen fibres with the typical zonal stratification of hyaline cartilage and has lower mechanical properties without complete integration and bonding of the collagen fibrils with the surrounding, unaffected cartilage. Often, the cartilaginous repair tissue within the defect shows signs of degeneration over time with matrix depletion, fragmentation and fibrillation.

The sequence of bone regeneration in osteochondral defects is different to that of the cartilage. In the deeper bony region, MSCs differentiate into osteocytes to form bone, probably via a process similar to the endochondral ossification seen during fracture healing [81]. This formation of immature bone usually restores the subchondral bone to its original level in a distinct pattern [66]. Such bone formation is associated with enhanced expression levels of the Runt‐related transcription factor 2 [6], a factor required for osteoblast differentiation that also regulates chondrocyte maturation and terminal differentiation and of type‐X collagen. Of note, the expression of SOX9 is absent. Over time, the front of this new subchondral bone may advance towards the joint space, and intralesional osteophytes may form within [69]. The cell types in the bony part of an osteochondral defect that sense and transduce mechanical stresses into a biological response include MSCs as osteoprogenitor cells, MSC‐derived osteoblasts that form the new subchondral bone, osteocytes (when terminally differentiated and embedded within the subchondral bone) and bone‐resorbing osteoclasts (derived from the macrophage lineage of hematopoietic stem cells). Especially osteocytes located within the newly formed subchondral bone plate bone sense external loads, followed by an adaptation of the architecture of the subchondral bone and an overall remodelling process that results in an advancement of the subchondral bone into the articular cartilage repair tissue.

Translational studies shed light on the interplay between cartilage and bone regeneration, revealing a defined chronological order for the reconstitution of the subchondral bone within osteochondral defects, which often results in the expansion of the subchondral bone plate into the cartilaginous repair tissue over time [66]. At the same time, cartilage repair within such defects is initially improved but later degraded, indicating the current inability of the repair tissues for true osteochondral regeneration. Interestingly, while individual parameters of subchondral bone and cartilage repair internally correlate with each other, no correlation was detected so far between the phenomenon of subchondral bone plate migration and the degradation of the repair cartilage in animal models [67]. This finding suggests that the advancement of the subchondral bone plate may not be responsible for the degradation of the cartilaginous repair tissue over time [66, 68].

Studies of human OA based on human tibial plateau samples revealed that in the subchondral bone in knee OA, bone matrix microdamage and vascular changes characterize the BMLs [65]. A study on BMLs in human knee OA investigated how subchondral bone microstructure within BMLs relates to the subchondral bone and cartilage across the human tibial plateau [64]. BMLs in OA were associated with increased thickness of the subchondral bone plate and trabecular number and separation. In established knee OA, both the extent of articular cartilage damage and the microstructural degeneration of the subchondral bone were dependent on the presence of a BML. More specifically, such subchondral BMLs are characterized by a greater heterogeneity of the bone mineralization density distribution and a low bone matrix mineralization [51].

Taken together, the osteochondral unit is regulated by cellular interactions, including osteocyte control of anabolic and catabolic turnover, signalling between MSCs, chondrocytes, osteoblasts, osteocytes and osteoclasts, and interaction with the cells from the adjacent articular cartilage and subchondral bone. This process is complex and may involve several, currently unknown physiological pathways. Research that aims at further elucidating the mechanisms of BML occurrence and clinical consequences is therefore of crucial importance and will help target better the different conservative approaches to address conservative BMLs.

## CONCLUSIONS

The management of BMLs is challenging, and many factors influence clinical and radiological outcomes. This paper summarized the available evidence on conservative treatments for knee BMLs, with positive results documented for unloading knee braces, ESWT, HBOT, PEMFs and BPs. Nonetheless, the analysis of the scientific literature documented a scarce number of publications specifically addressing the knee joint, with even less evidence when it comes to the results for the different aetiologies of BMLs. Although showing promising results, conservative options still need to be fully investigated. Open questions to be addressed concern treatment duration, BML stage and overlap with concomitant physical or pharmaceutical therapies. Finally, further studies are needed to identify the best first‐line conservative approach or treatment combination based on each BML aetiology.

## AUTHOR CONTRIBUTIONS

**Giuseppe Filardo**: Conceptualization; supervision; writing—review and editing. **Luca Andriolo**: Conceptualization; writing—review and editing. **Massimo Berruto**: Conceptualization; writing—original draft preparation. **Alessandro Sangiorgio**: Writing—original draft preparation. **Massimo Varenna**: Writing—original draft preparation. **Stefano Zaffagnini**: Writing—original draft preparation. **Beer Yiftah**: Writing—original draft preparation. **Henning Madry**: Writing—original draft preparation. **Giuseppe M. Peretti**: Writing—original draft preparation. All authors have read and agreed to the published version of the manuscript.

## CONFLICT OF INTEREST STATEMENT

The authors declare no conflicts of interest.

## ETHICS STATEMENT

No ethical committee approval or patient consent was needed due to the nature of the study.

## Data Availability

Data Availability Statement is not available.
